# Risk factors and prognostic predictors of recurrent bacterial empyema in patients after surgical treatment

**DOI:** 10.1186/s12879-025-11077-0

**Published:** 2025-05-06

**Authors:** Chia-Chi Liu, Yi-Ling Chen, Ching-Yuan Cheng, Chang-Lun Huang, Wei-Heng Hung, Bing-Yen Wang

**Affiliations:** 1 Division of Thoracic Surgery, Department of Surgery, Changhua Christian Hospital, Changhua City, Changhua County, Taiwan; 2 Surgery Clinical Research Center, Changhua Christian Hospital, Changhua City, Changhua County Taiwan; 3 Department of Post-Baccalaureate Medicine, College of Medicine, National Chung Hsing University, Taichung City, Taiwan

**Keywords:** Empyema thoracis, Recurrence, Video-assisted thoracoscopic surgery, Prognostic factors, Surgical outcomes

## Abstract

**Background:**

Thoracic empyema is a severe infection with significant morbidity and mortality. Recurrence remains a challenge, impacting patient outcomes and healthcare costs. This study investigated the incidence and prognostic factors of empyema recurrence to enhance management strategies.

**Methods:**

A retrospective cohort study was conducted on 1,000 patients over 18 years old with stage II or III thoracic empyema who underwent video-assisted thoracoscopic surgery (VATS) decortication between 2011 and 2022 at Changhua Christian Hospital in Taiwan. We excluded patients who had non-bacterial empyema. We also excluded those who experienced contralateral or same-admission recurrences. Clinical data were analyzed to identify factors associated with recurrence and outcomes.

**Results:**

Empyema recurred in 46 patients (4.6%), with a median recurrence time of 37.5 days. The recurrent group had higher rates of diabetes mellitus (47.8% vs. 31.7%, *p* = 0.022), stage III empyema (32.6% vs. 20.1%, *p* = 0.041), and pleural glucose levels ≤ 40 mg/dL (52.4% vs. 36.9%, *p* = 0.043). *Streptococcus* species infections were more prevalent among recurrent cases (*p* = 0.029). Delays from diagnosis to operation were longer in the recurrent group (10.46 ± 23.46 days). Recurrent patients experienced extended postoperative antibiotic use and longer intensive care unit stays and hospital admissions. Overall survival was significantly lower in the recurrent group during long-term follow-up (*p* < 0.001).

**Conclusions:**

Empyema recurrence after VATS decortication worsens outcomes. Key risk factors include diabetes mellitus, low pleural glucose levels, *Streptococcus* infection, and delayed surgery. Early diagnosis, prompt surgical intervention, and careful management of comorbidities may be beneficial to reduce recurrence and improve patient outcomes.

## Background

Thoracic empyema is increasingly acknowledged as a severe infectious condition globally. Recent epidemiological analyses indicate a clear increasing trend in empyema thoracis among United States adults through the 2010s. For example, a 2021 study of national hospital data found the empyema hospitalization rate rose from about 8.1 per 100,000 adults in 2007 to 11.1 per 100,000 in 2016 [1] Early adoption of minimally‑invasive (video‑assisted) drainage and faster referral have reduced operative mortality in adults from historical figures near 20% to < 10%. Large series show video-assisted thoracoscopic surgery (VATS) feasibility in > 80% of cases, with death now mainly linked to pre‑operative sepsis and severe comorbidity rather than age or procedure‑related factors [2, 3]. 

According to the 2017 American Association for Thoracic Surgery guidelines, empyema is categorized into three stages: the early exudative phase (stage I), the intermediate fibroproliferative phase (stage II), and the organizing phase (stage III) [4]. In stage I, surgical intervention and chest tube drainage are generally unnecessary [5]. For stages II and III, closed drainage and decortication are strongly recommended. Recent research indicates that VATS achieves comparable surgical outcomes to traditional methods while reducing postoperative complications [6–9]. 

Empyema thoracis could correlate with many complications such as sepsis, respiratory failure, lung atelectasis, and bronchopleural fistulas. These complications may lead to a prolonged hospital stay time and mortality; they thus affect patients’ quality of life and increase healthcare costs [10]. The recurrence of empyema could be regarded as a failure of treatment; it was first reported in 1.6% of acute and chronic empyema patients by Meade in 1935 [11]. Despite the advance of surgical intervention with VATS decortication in the past decade, the recurrence rate of empyema still varies from 0.93 to 4.8% [12–15]. 

Despite recent therapeutic progress, the determinants of postoperative recurrence of bacterial empyema are still unclear. Previous series did not specifically examine recurrence, even though it develops in roughly 1–5% of surgically treated patients and is linked to poorer long‑term outcomes. Defining these risk factors therefore constitutes the central rationale for the present study. We aimed to investigate the incidence of recurrent empyema in our institute and to identify the prognostic factors of the recurrence. Furthermore, we seek for the appropriate management to prevent recurrence and to reduce the mortality and morbidity rates of empyema.

## Methods

### Patient population and selection

A retrospective cohort study was conducted at Changhua Christian Hospital in Taiwan to analyze patients over 18 years old with phase II or III thoracic empyema who underwent video-assisted thoracoscopic pleural decortication from April 2011 to May 2022. Empyema thoracis was defined as pus in the pleural space, confirmed either by positive pleural effusion cultures or by imaging showing loculated parapneumonic effusion. From 1,086 identified patients, exclusion criteria were applied: respiratory secretion cultures yielding *Mycobacterium tuberculosis* or fungi (*n* = 77), recurrence of empyema in the contralateral pleural space (*n* = 4), and recurrence diagnosed during the same hospital admission (*n* = 5). Patients with fungal empyema were excluded because they represented a very small subset and require antifungal protocols distinct from antibacterial management, and a parallel analysis in the same cohort showed markedly poorer surgical outcomes for fungal versus bacterial empyema, which would bias recurrence modelling [16]. After exclusions, 1,000 patients remained and were included in the study analysis.

### Clinical management and recurrence definition

When patients presented with airway infection symptoms, a chest X-ray (CXR) was arranged. If pleural effusion was detected, invasive procedures like thoracentesis or chest tube insertion were performed to collect effusion specimens for biochemical analysis and culture. Patients were admitted for empiric antibiotic therapy, monitoring of chest drainage, and continued CXR follow-ups. Antibiotics were adjusted based on sensitivity results from the specimens obtained. If pleural effusion persisted, computed tomography (CT) of the chest was performed. Loculated pleural effusion on imaging raised high suspicion for phase II/III empyema, indicating surgical intervention. Video-assisted thoracoscopic decortication was then performed, during which pleural fluid and pleural peel tissue cultures were obtained. One or two chest tubes were placed in the pleural space for adequate drainage. Postoperatively, persistent pleural space was evaluated via CXR three days to one week after surgery. Negative pressure suction (-15 cmH2O) was applied if poor lung expansion was observed with persistent pleural space or daily pleural effusion accumulation over 200 ml. The duration of antibiotic use and chest tube drainage depended on the patient’s clinical presentation and imaging findings. Patients were discharged once chest drainage could be removed and intravenous antibiotics were no longer needed. Follow-up visits at the thoracic surgery clinic included CXRs at 1 week, 1 month, and 3 months after surgery, then every 6 months. Recurrent empyema was defined as a new episode of purulent pleural infection on the same hemithorax occurring after complete clinical and radiographic resolution of the index empyema and after chest-tube removal or hospital discharge. Relapse had to meet all of the following criteria. (1) Compatible symptoms or signs, such as fever > 38 °C, leukocytosis, or pleuritic pain. (2) Imaging evidence of a pleural collection requiring intervention. (3) Intra-operative documentation of pus or a positive pleural fluid/tissue cultures (4) Necessity for repeat drainage (tube, VATS, or thoracotomy) within 90 days of the index surgery. The relapse was diagnosed when imaging indicated a new, loculated pleural collection ≥ 10 mm. On contrast-enhanced chest CT—the reference standard—this had to be accompanied by circumferential pleural thickening with an air-fluid level or by passive lung collapse of at least one segment. If CT was unavailable, bedside ultrasonography showing an anechoic or septated pocket ≥ 10 mm in two orthogonal planes was accepted; for screening radiographs, a vertical meniscus height ≥ 20 mm (about 200 mL) on an upright poster-anterior film triggered further cross-sectional imaging. Contralateral effusions and collections detected during the initial admission were not considered relapses and were excluded. If recurrent empyema was highly suspected during follow-up, further evaluation was conducted to confirm the diagnosis, and hospital admission was arranged.

### Clinical features and data collection

We analyzed patient characteristics including age, sex, smoking history, comorbidities, empyema location and phase, laboratory data, pleural fluid data, pathogens, antibiotic usage, length of ICU stay (ICLOS), length of hospital stay (HLOS), and mortality. Observations were defined according to the 2017 American Association for Thoracic Surgery consensus guidelines. Indicators of infectious pleural fluid were pH < 7.2, glucose < 40 mg/dL, and lactate dehydrogenase (LDH) > 1,000 IU/L. Since all patients underwent thoracoscopic decortication, we also analyzed surgery-related factors such as the duration between diagnosis and operation, duration between drainage and operation, postoperative drainage duration, and ICU and ward admission days. The primary outcome was empyema recurrence; secondary outcomes included mortality, hospital stay length, duration of chest drainage, and timing of operation. We define ‘delayed surgery’ as the initial management approach where, within 48 h following the diagnosis of empyema, only thoracentesis for pleural effusion drainage or chest tube placement is performed, without proceeding to decortication surgery.

### Statistical analysis

Patients were divided into two groups: recurrent and nonrecurrent. Clinical factors—including age, sex, smoking history, comorbidities, empyema location and phase, laboratory data, pleural fluid data, pathogens, antibiotic usage, ICLOS, HLOS, and mortality—were analyzed to identify prognostic factors for recurrence. The Mann-Whitney U test was used to evaluate continuous variables, while the chi-squared test compared categorical variables. A p-value less than 0.05 indicated statistical significance. Overall survivals were evaluated with Kaplan-Meier curves, and inter-group differences were tested with the log-rank test. Because postoperative recurrence is a binary outcome, we applied multivariate binary logistic regression. All demographic, laboratory, and peri-operative variables listed in Table 4 were first screened with univariate tests (Mann-Whitney U for continuous data, χ² or Fisher’s exact test for categorical data). Covariates showing *p* < 0.10 in this screen, together with clinically important factors specified a priori (age, sex, symptom-to-surgery interval), were entered into the multivariate model. Multicollinearity was assessed by variance-inflation factors and tolerance statistics. A backward step-wise likelihood-ratio procedure (removal threshold *p* > 0.15) produced the final model, which identified independent predictors of recurrence and provided adjusted odds ratios with 95% confidence intervals. All statistical analyses were performed using SPSS software (version 23.0; SPSS Inc., Chicago, IL, USA).

## Results

In this study of 1,000 empyema patients, 46 cases (4.6%) experienced recurrence, with a median recurrence time of 37.5 days. Table 1  shows significant clinical differences between the recurrent and nonrecurrent groups. Diabetes mellitus was more prevalent in the recurrent group (47.8%) compared to the nonrecurrent group (31.7%) (*p* = 0.022), while no significant differences were found for liver cirrhosis, end-stage renal disease, or congestive heart failure. The nonrecurrent group had a higher proportion of phase II empyema (79.9%) versus the recurrent group (67.4%), whereas phase III empyema was more common in the recurrent group (32.6%, *p* = 0.041). Pleural glucose levels ≤ 40 mg/dL were significantly higher in the recurrent group (52.4%) than in the nonrecurrent group (36.9%, *p* = 0.043). *Streptococcus* species prevalence differed significantly between groups, which increased from 202/954 (21.2%) to 16/46 (34.8%) (*p* = 0.029). Clinically, the recurrent group had a longer duration of postoperative antibiotic usage (18.11 ± 30.77 days vs. 29.83 ± 26.04 days, *p* < 0.001), and the nonrecurrent group had shorter intensive care unit (ICU) and hospital stays during the initial admission (*p* = 0.034 and *p* < 0.001, respectively). These findings suggest that various clinical and microbiological factors correlate with empyema recurrence, indicating the need for tailored clinical management to reduce recurrence risk and improve patient outcomes.

**Table 1:** Patient characteristics of recurrent and nonrecurrent empyema groups

Factors	Total cohort (*n* = 1000)	Nonrecurrent (*n* = 954)	Recurrent (*n* = 46) (first admission)	*p*-value
Age, Median (IQR) (y) ^a^	62.00(50.25-75.00)	62.00(51.00–75.00)	63.50(49.00–75.00)	0.917
Sex^b^				
Male	779(77.9%)	738(77.4%)	41(89.1%)	0.060
Female	221(22.1%)	216(22.6%)	5(10.9%)	
Smoking^b^	300(30.0%)	286(30.0%)	14(30.4%)	0.947
Comorbidity				
Malignancy^b^	287(28.7%)	268(28.1%)	19(41.3%)	0.053
DM^b^	324(32.4%)	302(31.7%)	22(47.8%)	0.022
HTN^b^	535(53.5%)	510(53.5%)	25(54.3%)	0.906
Liver cirrhosis^b^	78(7.8%)	73(7.7%)	5(10.9%)	0.397
ESRD^b^	84(8.4%)	78(8.2%)	6(13.0%)	0.245
CHF^b^	68(6.8%)	66(6.9%)	2(4.3%)	0.764
COPD^b^	195(19.5%)	184(19.3%)	11(23.9%)	0.439
Location^b^				
Right	595(59.5%)	568(59.5%)	27(58.7%)	0.729
Left	393(39.3%)	374(39.2%)	19(41.3%)	
Bilateral	12(1.2%)	12(1.3%)	0(0.0%)	
Phase^b^				
II	793(79.3%)	762(79.9%)	31(67.4%)	0.041
III	207(20.7%)	192(20.1%)	15(32.6%)	
Lab data				
WBC, Median (IQR), (10^3^/µL) ^a^	13.00(9.80–17.90)	13.00(9.80–18.00)	13.25(8.58–17.30)	0.382
ANC, Median (IQR), (10^3^/µL) ^a^	10.59(7.57–15.32)	10.57(7.64–15.38)	11.29(6.40-15.19)	0.593
Pleural data				
Pleural pH ( ≦ 7.2) ^b^	421(48.3%)	399(47.8%)	22(57.9%)	0.225
Pleural glucose ( ≦ 40 mg/dL) ^b^	338(37.6%)	316(36.9%)	22(52.4%)	0.043
Pleural LDH ( ≧ 1000 IU/L) ^b^	541(60.4%)	518(60.7%)	23(56.1%)	0.560
Pathogen				
*Staphylococcus aureus*^b^	61(6.1%)	56(5.9%)	5(10.9%)	0.194
*Streptococcus* spp. ^b^	218(21.8%)	202(21.2%)	16(34.8%)	0.029
PsA^b^	36(3.6%)	32(3.4%)	4(8.7%)	0.078
*Klebsiella pneumoniae*^b^	85(8.5%)	78(8.2%)	7(15.2%)	0.102
A.B. ^b^	15(1.5%)	14(1.5%)	1(2.2%)	0.509
MRSA^b^	31(3.1%)	28(2.9%)	3(6.5%)	0.167
1st admission post-op ^a^ antibiotics usage (days) ^a^	11.00(8.00–20.00)	11.00(8.00–19.00)	21.50(11.00-36.75)	< 0.001
1st admission length of ICU admission ^a^	0.00(0.00–10.00)	0.00(0.00–9.00)	6.00(0.00–15.00)	0.034
1st admission length of hospital stay ^a^	16.50(11.00–30.00)	16.00(11.00–29.00)	32.00(15.00-58.50)	< 0.001
In-hospital mortality^b^	100(10.0%)	92(9.6%)	8(17.4%)	0.087
30-day mortality^b^	58(5.8%)	57(6.0%)	1(2.2%)	0.513

ANC, absolute neutrophil count; CHF, congestive heart failure; COPD, chronic obstructive pulmonary disease; DM, diabetes mellitus; ESRD, end-stage renal disease; HTN, hypertension; LDH, lactate dehydrogenase; MRSA, methicillin-resistant *Staphylococcus aureus*; WBC, white blood count

a: Mann-Whitney U test, b:chi-squared test or Fisher exact test

All empyema patients in the study underwent thoracoscopic decortication at least once. In Table 2, we compared nonrecurrent and recurrent groups regarding surgical intervention factors. Patients with recurrent thoracic empyema experienced significantly longer durations between diagnosis and operation (10.46 ± 23.46 days) and between initial chest drainage and operation (3.09 ± 5.28 days) compared to nonrecurrent patients. Additionally, the recurrent group had extended postoperative drainage durations (33.52 ± 48.22 days), longer postoperative ward admissions (41.89 ± 50.82 days), and increased postoperative ICU admissions (8.87 ± 15.78 days). These findings highlight the greater complexity and prolonged recovery associated with managing patients who experience recurrences of thoracic empyema.

**Table 2:** Comparison of surgical intervention between groups (1st admission)

Factors	Total cohort (*n* = 1000)	Nonrecurrent (*n* = 954)	Recurrent (*n* = 46)	*p*-value
Duration between diagnosis and OP (days)^a^	3.00(0.25-6.00)	2.00(0.00–6.00)	5.00(2.00–11.00)	0.002
Duration between drainage and OP (days) ^a^	0.00(0.00–2.00)	0.00(0.00–2.00)	0.00(0.00–5.00)	0.005
Post-OP drainage duration (days) ^a^	8.00(6.00–13.00)	8.00(6.00–13.00)	15.00(9.00-30.50)	< 0.001
1 chest tube^b^	56(5.6%)	51(5.3%)	5(10.9%)	0.120
2 chest tubes	925(92.5%)	886(92.9%)	39(84.8%)	
3 chest tubes	17(1.7%)	15(1.6%)	2(4.3%)	
4 chest tubes	2(0.2%)	2(0.2%)	0(0.0%)	
Pre-Op ward admission (days) ^a^	3.00(1.00–7.00)	3.00(1.00–7.00)	5.00(1.75–10.25)	0.058
Pre-Op ICU admission (days) ^a^	0.00(0.00–0.00)	0.00(0.00–0.00)	0.00(0.00-3.25)	0.071
Post-Op ward admission (days) ^a^	12.00(8.00–21.00)	11.00(8.00–21.00)	24.00(11.00-42.75)	< 0.001
Post-Op ICU admission (days) ^a^	0.00(0.00–7.00)	0.00(0.00–6.00)	4.00(0.00-12.50)	0.022

ICU, intensive care unit

a: Mann-Whitney U test, b:chi-squared test or Fisher exact test

To compare initial and recurrent episodes in the recurrent empyema group, we analyzed clinical parameters of these 46 patients (Table 3). Initially, 67.4% were diagnosed with stage II empyema, but during recurrence, 65.2% were diagnosed with stage III empyema (*p* = 0.002). There was no significant difference in lab data, pleural effusion analysis, or culture results between episodes. Only the prevalence of *Streptococcus* species was significantly higher during the first admission (16/46, 34.8%) than the second admission (5/46, 10.9%) (*p* = 0.006). More methicillin-resistant *Staphylococcus aureus* (MRSA) was found during the second admission (5/46, 10.9%) compared to the first (3/46, 6.5%), but this increase was not statistically significant.

**Table 3:** Patient characteristics in the recurrent group stratified by admission (*n* = 46)

	1st admission	2nd admission	*p*-value
Stage^b^			
II	31(67.4%)	16(34.8%)	0.002
III	15(32.6%)	30(65.2%)	
Lab data			
WBC, Mean ± SD (10^3^/µL) ^a^	13.25(8.58–17.30)	11.90(7.35–15.88)	0.550
ANC, Mean ± SD (10^3^/µL) ^a^	11.29(6.40-15.19)	10.64(5.26-14.00)	0.493
Pleural data			
Pleural pH ( ≦ 7.2) ^b^	22(57.9)	17(65.4%)	0.609
Pleural glucose ( ≦ 40 mg/dL) ^b^	22(52.4%)	13(50.0%)	0.849
Pleural LDH ( ≧ 1000 IU/L) ^b^	23(56.1%)	17(65.4%)	0.450
Culture results^b^			
Negative	12(26.1%)	14(30.4%)	0.643
Positive	34(73.9%)	32(69.6%)	
Blood culture^b^	5(10.9%)	7(15.2%)	0.536
Pleural effusion or tissue culture^b^	33(71.7%)	28(60.9%)	0.270
Single pathogen	10(50.0%)	7(53.8%)	0.829
Multiple pathogen	10(50.0%)	6(46.2%)	
Pathogen			
*Staphylococcus aureus*^b^	5(10.9%)	5(10.9%)	> 0.999
*Streptococcus* spp. ^b^	16(34.8%)	5(10.9%)	0.006
PsA^b^	4(8.7%)	3(6.5%)	> 0.999
*Klebsiella pneumoniae*^b^	7(15.2%)	7(15.2%)	> 0.999
A.B. ^b^	1(2.2%)	1(2.2%)	> 0.999
MRSA^b^	3(6.5%)	5(10.9%)	0.714

ANC, absolute neutrophil count; LDH, lactate dehydrogenase; MRSA, methicillin-resistant *Staphylococcus aureus*; WBC, white blood count

a: Mann-Whitney U test, b:chi-squared test or Fisher exact test

**Fig. 1:**
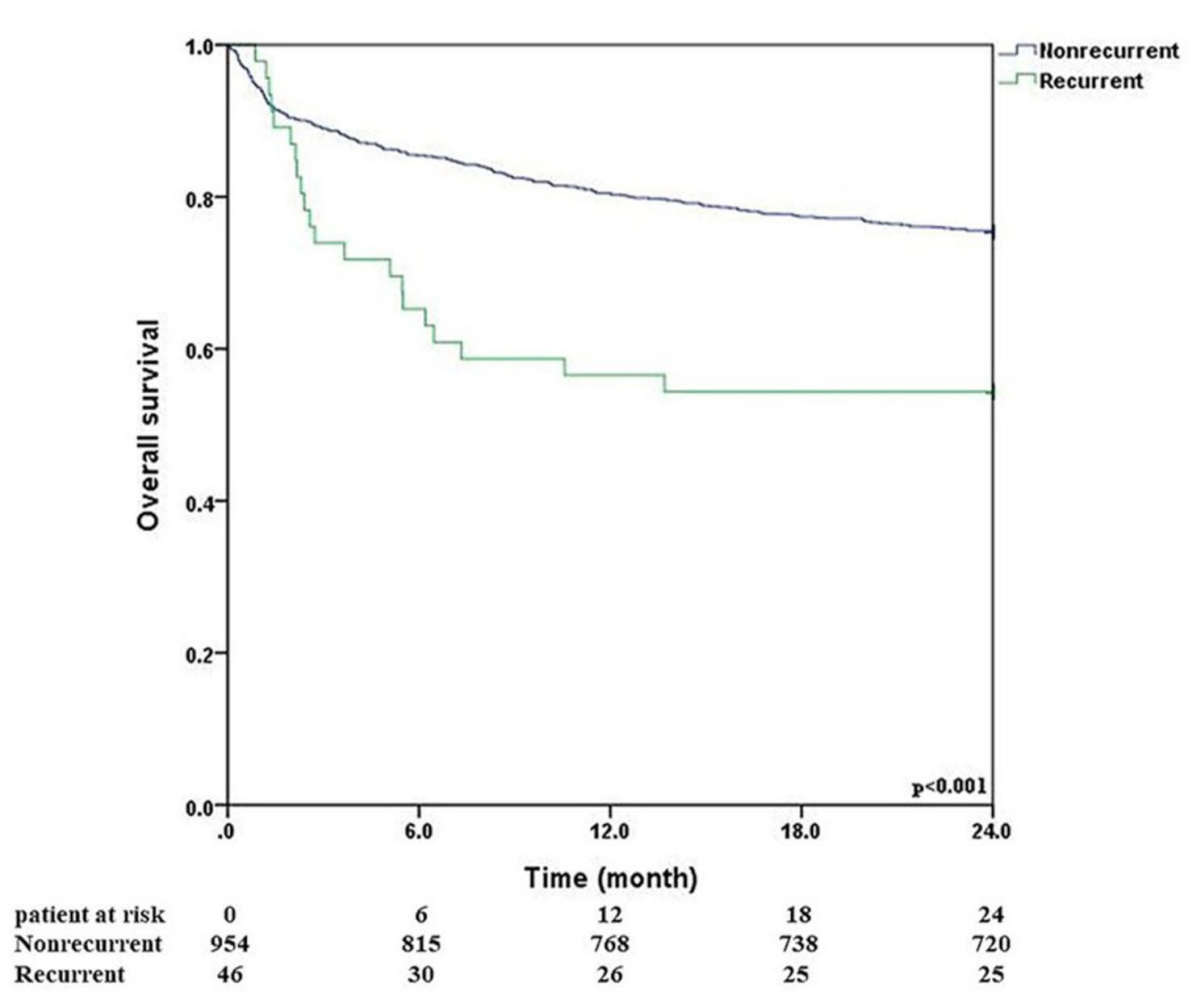
Kaplan–Meier curve with the log-rank test illustrating overall survival for the recurrent and nonrecurrent groups

Concerning the primary outcome, the overall survival rate did not differ between the recurrent and nonrecurrent groups while limiting the duration to the hospital stay or to 30 days. However, a difference in overall survival between the two groups became apparent during 6-month, 12-month, 18-month and 24-month follow-ups (*p* < 0.001) (Fig. 1). We conducted univariable and multivariable analyses and found that several factors were significantly associated with higher recurrent rate in thoracic empyema patients undergoing video-assisted thoracoscopic decortication, as can be seen in Table 4. Diabetes mellitus was a significant risk factor (multivariable odds ratio (OR): 2.25, *p* = 0.035). In univariable analysis, patients with stage III empyema had higher recurrent rate compared to those with stage II empyema (OR: 1.92, *p* = 0.044). Low pleural glucose levels (≤ 40 mg/dL) were linked to increased recurrent rate (multivariable OR: 2.38, *p* = 0.048). Additionally, the presence of *Streptococcus* spp. significantly increased the risk of recurrence (multivariable OR: 2.66, *p* = 0.009). As a result, intensive control of hyperglycemia and administration of empiric antibiotics for *Streptococcus* infections were the strategies to be considered for preventing recurrent empyema.

**Table 4:** Univariable and multivariable analyses of factors affecting recurrence of empyema

	Univariable		Multivariable	
	Odds Ratio (95% CI)	*p*-value	Odds Ratio (95% CI)	*p*-value
Age	1.00(0.99–1.02)	0.825	1.00(0.97–1.02)	0.766
Sex				
Male	2.4(0.94–6.15)	0.068	2.08(0.68–6.35)	0.196
Female	1		1	
Smoking	1.02(0.54–1.94)	0.947	0.81(0.38–1.77)	0.603
Comorbidity				
Malignancy	1.80(0.99–3.29)	0.056	1.44(0.69–2.97)	0.329
DM	1.98(1.09–3.59)	0.024	2.25(1.06–4.79)	0.035
HTN	1.04(0.57–1.88)	0.906	0.80(0.37–1.72)	0.558
Liver cirrhosis	1.47(0.56–3.84)	0.429	1.05(0.33–3.32)	0.931
ESRD	1.69(0.69–4.10)	0.250	1.83(0.62–5.40)	0.276
CHF	0.61(0.15–2.58)	0.503	0.30(0.04–2.39)	0.254
COPD	1.32(0.66–3.64)	0.441	1.23(0.53–2.88)	0.632
Location				
Right	1		1	
Left or Bilateral	1.04(0.57–1.89)	0.909	1.07(0.54–2.14)	0.845
Phase				
II	1		1	
III	1.92(1.02–3.63)	0.044	1.48(0.68–3.25)	0.327
Lab data				
WBC (10^3^/µL)	0.97(0.93–1.02)	0.275	0.96(0.86–1.07)	0.468
ANC (10^3^/µL)	0.98(0.93–1.03)	0.468	1.03(0.94–1.14)	0.513
Pleural data				
Pleural pH ( ≦ 7.2)	1.50(0.78–2.90)	0.228	1.05(0.48–2.29)	0.900
Pleural glucose ( ≦ 40 mg/dL)	1.88(1.01–3.50)	0.046	2.38(1.01–5.64)	0.048
Pleural LDH ( ≧ 1000 IU/L)	0.83(0.44–1.56)	0.560	0.44(0.19–1.01)	0.052
Pathogen				
*Staphylococcus aureus*	1.96(0.74–5.14)	0.174	2.44(0.51–11.69)	0.265
*Streptococcus* spp.	1.99(1.06–3.71)	0.032	2.66(1.27–5.55)	0.009
PsA	2.74(0.93–8.12)	0.068	2.70(0.71–10.17)	0.143
*Klebsiella pneumoniae*	2.02(0.87–4.66)	0.101	1.67(0.52–5.35)	0.385
A.B.	1.49(0.19–11.60)	0.702	0.78(0.08–7.17)	0.824
MRSA	2.31(0.68–7.89)	0.183	1.01(0.11–9.02)	0.990

ANC, absolute neutrophil count; CHF, congestive heart failure; COPD, chronic obstructive pulmonary disease; DM, diabetes mellitus; ESRD, end-stage renal disease; HTN, hypertension; LDH, lactate dehydrogenase; MRSA, methicillin-resistant *Staphylococcus aureus*; WBC, white blood count

Logistic regression

**Fig. 2:**
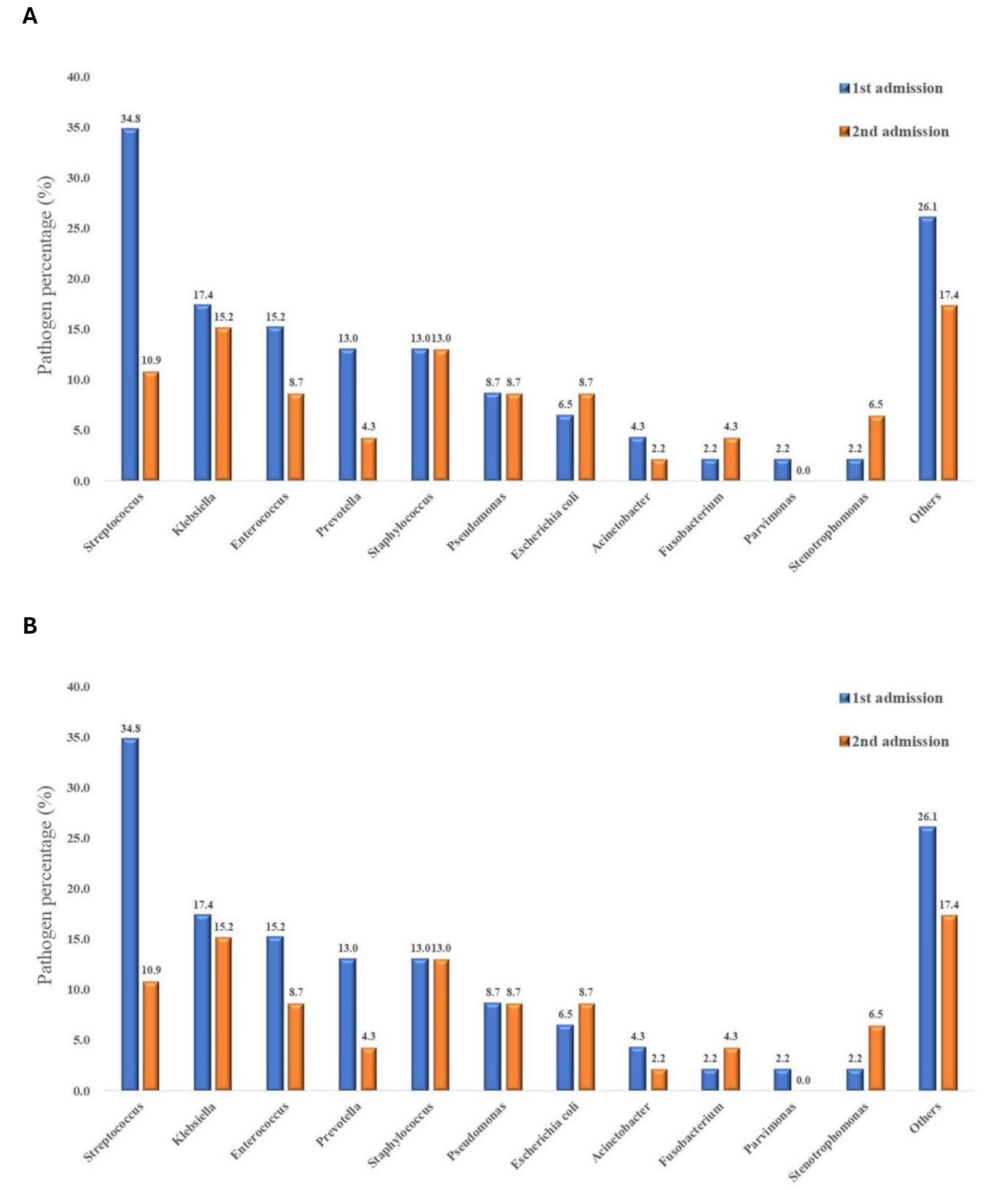
Culture positive rates of various pathogens: **2-A.** Within the recurrent and nonrecurrent groups. **2-B.** Based on the first and second admissions within the recurrent group

In Fig. 2-A, we compare the culture-positive rates of common pathogens between recurrent and nonrecurrent groups. During the first admissions, *Streptococcus* species were the most common pathogen in both groups but were more prevalent in the recurrent group (34.8%) than in the nonrecurrent group (21.2%). Among recurrent patients, pathogen profiles differed between first and second admissions (Fig. 2-B). During the second admission, the most common pathogen was *Klebsiella pneumoniae*, followed by *Staphylococcus aureus* and *Streptococcus* spp. Overall, the positive culture rate during the second admission was lower than during the first admission. These findings indicate a shift in pathogen prevalence in recurrent empyema cases and a general decrease in positive culture rates over time.

In this study, missing data were minimal for most variables, with missing rates as follows: White blood cell (WBC) (1.5%), absolute neutrophil count (ANC) (1.9%), Pleural glucose ( ≦ 40 mg/dL) (10.2%), and Pleural LDH ( ≧ 1000 IU/L) (10.5%). The categorical variable Pleural pH ( ≦ 7.2) had a slightly higher missing rate of 12.8%. Although this variable exhibited a higher proportion of missing data, it was neither a primary predictor nor an outcome variable, and its impact on the statistical results was deemed minimal. Given that Pleural pH is derived from intraoperative specimen testing and serves as an indicator in empyema guidelines, imputation methods were considered inappropriate for estimating missing values. To ensure the validity of statistical inferences, a conservative approach was adopted, and complete case analysis was used for handling missing data.

## Discussion

This single-center study analyzed 1,000 patients with bacterial empyema thoracis who underwent VATS decortication. We reported 46 cases of recurrence (4.6%), with a median time to recurrence of 37.5 days. Patients with recurrent empyema indicated a lower overall survival rate after 6 months. Additionally, the recurrent group experienced longer postoperative antibiotic use, extended ICU stays, and longer hospital admissions during the initial admissions. Factors such as underlying diabetes mellitus, lower glucose levels in pleural effusion, and *Streptococcus*-related infections may be associated with recurrence. Furthermore, we found that the duration between diagnosis and operation was significantly longer in the recurrent group, as was the duration between chest drainage and operation. These findings suggest that early intervention with VATS decortication may effectively reduce the risk of empyema recurrence.

Our study identified diabetes mellitus as a significant risk factor for the recurrence of thoracic empyema, with a higher prevalence in the recurrent group compared to the nonrecurrent group (47.8% vs. 31.7%, *p* = 0.022). This aligns with existing literature indicating that diabetes mellitus predisposes individuals to infections due to immune dysfunction associated with hyperglycemia and impaired neutrophil function [17]. Specifically, in empyema thoracis, diabetic patients have been reported to experience higher morbidity and mortality rates [18]. Retrospective studies suggested that diabetes mellitus patients with pleural infections had prolonged hospital stays and increased complication rates [19, 20]. Therefore, stringent glycemic control and early intervention are crucial in diabetic patients to reduce the risk of empyema recurrence and improve clinical outcomes.

The decision to use a pleural fluid glucose cut-off of ≤ 40 mg/dL is supported by converging evidence. First, major guidelines from the American College of Chest Physicians, British Thoracic Society, and American Association for Thoracic Surgery uniformly designate 40 mg/dL (2.2 mmol/L) as the critical threshold below which a parapneumonic effusion is classified as complicated and warrants prompt intercostal or surgical drainage [4, 5, 21]. Second, pathophysiologically, glucose concentrations fall as bacteria and activated leukocytes consume substrate or as pleural thickening impedes diffusion; levels < 60 mg/dL are sensitive but less specific, whereas values ≤ 40 mg/dL correlate most strongly with high bacterial burden, low pH and subsequent need for invasive therapy [22]. Finally, prospective data suggest that glucose ≤ 40 mg/dL independently predicts failure of antibiotic therapy and worse surgical outcomes, underscoring its prognostic value [23]. Adopting this stricter cut-off therefore aligns our analysis with contemporary standards while ensuring a clinically homogeneous high-risk cohort for modelling recurrence factors. Elevated lactate dehydrogenase (LDH) and protein levels in pleural fluid reflect increased metabolic activity of bacteria and leukocytes within the pleural space [24]. In our analysis, pleural glucose levels ≤ 40 mg/dL were significantly higher in the recurrent group (52.4%) than in the nonrecurrent group (36.9%) (*p* = 0.043). Our findings support the use of pleural fluid analysis, including glucose, LDH, and protein levels, as valuable tools in predicting severity and guiding the management of empyema thoracis.

Geography and care setting dictate the microbiology of pleural infection. A 10,000-case systematic review showed Staphylococcus aureus to be the chief pathogen worldwide, whereas pneumococci and viridans streptococci dominate in tropical regions such as Taiwan [20]. In our cohort, Streptococcus spp. were indeed common on first admission and were significantly more frequent in patients who later relapsed (34.8% vs. 21.2%; *p* = 0.029). At recurrence, however, the spectrum shifted toward Klebsiella pneumoniae and S. aureus (including MRSA), mirroring East-Asian ICU reports in which prior antibiotic exposure, diabetes and healthcare contact favor Gram-negative bacilli and drug-resistant staphylococci [25]. Recognizing these predictable, region-specific transitions is essential for selecting empirical antibiotics that both clear the initial infection and minimise the risk of relapse.

Patients who ultimately required re-operation tended to undergo an initial “conservative window” in which we attempted tube drainage and antibiotics before taking them back to theatre. Dense adhesions, pleural peel maturation and the technical difficulty of a second decortication all favour a brief trial of non-operative therapy first. We therefore defined delayed surgery as an index management strategy in which, within 48 h of radiologic diagnosis of empyema, only thoracentesis or chest-tube drainage was performed and no decortication (VATS or open) was undertaken. Cases proceeding directly to VATS decortication inside that 48-h window were classed as timely surgery. In the sole randomised trial comparing early VATS with chest-tube ± streptokinase, Wait et al. showed a primary success rate of 91% vs. 44%, shorter chest-tube duration (5.8 vs. 9.8 days) and a 4-day reduction in length of stay for the early-VATS group; all medical failures eventually required surgery [26]. Similarly, Chen et al. reported that video-assisted decortication performed after a prolonged disease course (> 1 month, “stage III”) carried a nine-fold higher complication rate (44% vs. 4.8% for stage I–II) and more incomplete lung re-expansion than early intervention [27]. These data support our finding that the recurrent cohort—who had a median diagnosis-to-operation interval of 10.5 days—fared worse: the delay allowed organization of the pleural peel, making subsequent surgery less effective and raising the risk of residual loculations that seed relapse. Our definition of “delayed” (> 48 h without decortication) therefore captures the clinically relevant threshold beyond which outcomes demonstrably deteriorate and aligns with evidence favoring prompt VATS once tube drainage fails to achieve rapid clearance. Recurrent empyema patients had significantly longer postoperative antibiotic usage and extended ICU and hospital stays. Moreover, the overall survival rate was markedly lower in the recurrent group during follow-ups at 6, 12, 18, and 24 months (*p* < 0.001). Recurrent empyema is associated with increased morbidity and poses substantial challenges in management [28]. Research suggests that inadequate initial treatment, resistant pathogens, and patient comorbidities contribute to recurrence [29]. These findings highlight the need for comprehensive initial management strategies to prevent recurrence and improve long-term outcomes.

Besides diabetes mellitus and low pleural glucose levels, our study identified the presence of *Streptococcus* species and delayed surgical intervention as significant predictors of empyema recurrence. Additionally, in the recurrent group the rate of stage III empyema increased from 32.6% among the initial empyema episodes to 65.2% during the recurrence episodes (*p* = 0.002), indicating disease progression. Advanced empyema stages are associated with fibrothorax and lung entrapment, complicating surgical management [30]. Factors such as advanced age, malnutrition, and immunosuppression have also been reported to influence recurrence and mortality rates [31]. Therefore, identifying high-risk patients through these predictors allows for tailored interventions aimed at reducing the recurrence of empyema and improving survival rates.

Our study has several limitations. First, being a retrospective single-center study, there is potential for selection bias, and the findings may not be generalizable to other institutions or populations with different demographics or healthcare practices. Second, the relatively small number of recurrent cases (46 out of 1,000 patients) may limit the statistical power of our analyses and the ability to detect subtle associations or draw definitive conclusions about risk factors for recurrence. Third, variations in surgical techniques, perioperative care, and antibiotic regimens over the extended study period (April 2011 to May 2022) could have influenced patient outcomes, but these were not controlled for in our analysis. Fourth, unmeasured confounding factors, such as patient adherence to postoperative care instructions, nutritional status, and socioeconomic status, were not accounted for and may have impacted recurrence rates and overall survival. Fifth, the follow-up period, while sufficient to capture most recurrences, may not have been long enough to detect late recurrences in some patients. Sixth, differences in management protocols and diagnostic criteria over time could have introduced inconsistencies in data collection and interpretation. As the surgery, several clinically relevant variables were not captured in our dataset and could have influenced both recurrence and survival. Although every procedure was labelled “two-port VATS decortication,” nuances in surgical execution (extent of peel removal, irrigation volume, postoperative suction settings, use of adjunctive fibrinolytics) inevitably varied among surgeons and may have affected residual pleural space and relapse risk. We likewise lacked objective measures of postoperative adherence—duration and timing of oral antibiotics, engagement with chest physiotherapy, and compliance with scheduled follow-up—which can modulate infectious control and functional recovery. Finally, nutritional status was approximated only by admission albumin; more sensitive indices (e.g., pre-albumin, Body Mass Index trends) were unavailable for most patients. Malnutrition impairs immunity and tissue healing and therefore represents a potential confounder in the observed link between delayed surgery, diabetes and recurrence. Despite these limitations, our study provides valuable insights into the risk factors associated with recurrent empyema thoracis and underscores the importance of early surgical intervention and meticulous management of comorbid conditions like diabetes mellitus. Prospective multicenter investigations are therefore required to confirm the associations we observed and to refine empiric-treatment recommendations.

## Conclusions

In conclusion, our study indicated that the recurrence rate of thoracic empyema after VATS decortication is relatively low at 4.6%, but recurrence is associated with significantly worse outcomes, including longer hospital stays, prolonged antibiotic use, and decreased overall survival during long-term follow-up. Key risk factors identified for recurrence include diabetes mellitus, low pleural glucose levels, infection with *Streptococcus* species, and delayed surgical intervention. These findings underscore the importance of early diagnosis and prompt surgical management, as well as meticulous control of comorbid conditions like diabetes, to reduce the risk of empyema recurrence and improve patient outcomes. Implementing tailored clinical strategies for high-risk patients may help in decreasing morbidity and enhancing survival rates. Further prospective, multicenter studies are recommended to validate these results and to develop comprehensive guidelines for managing empyema thoracis effectively.

## Data Availability

The datasets generated and/or analysed during the current study are not publicly available due to the privacy of individuals that participated in the study but are available from the corresponding author on reasonable request.

## Abbreviations

ANC: Absolute neutrophil count
CHF: Congestive heart failure
COPD: Chronic obstructive pulmonary disease
CXR: Chest X-ray
DM: Diabetes mellitus
ESRD: End-stage renal disease
HLOS: Length of hospital stay
HTN: Hypertension
ICLOS: Length of intensive care unit stay
ICU: Intensive care unit
LDH: Lactate dehydrogenase
MSRA: Methicillin-resistant *Staphylococcus aureus*
OR: odds ratio
VATS: Video-assisted thoracoscopic surgery
WBC: White blood count
